# Advancing Stepped-Waveform Radar Jamming Techniques for Robust False-Target Generation against LFM-CFAR Systems

**DOI:** 10.3390/s23187782

**Published:** 2023-09-10

**Authors:** Yanqi Wang, Chao Wang, Qingzhan Shi, Jingjian Huang, Naichang Yuan

**Affiliations:** 1 College of Electronic Science and Technology, National University of Defense Technology, Changsha 410073, China; 2 State Key Laboratory of Complex Electromagnetic Environment Effects on Electronics and Information System, National University of Defense Technology, Changsha 410073, China

**Keywords:** radar jamming, false target generation, stepped waveform modulation, linear frequency modulation, constant false alarm rate detection

## Abstract

This study investigates the utilization of a stepped wave frequency modulation jamming technique in radar systems. The objective is to enhance the effectiveness and robustness of false target jamming in the presence of linear frequency modulation (LFM) radars employing constant false alarm rate (CFAR) detection. The proposed method combines stepped frequency modulation with full pulse delay/sum repeat jamming to enhance resilience against uncertainties in target parameters. Theoretical analysis and simulation experiments are conducted to establish relationships between key jammer parameters, such as frequency slope and power compensation, and performance metrics, like false target distribution and CFAR masking. The results demonstrate that the proposed technique effectively maintains a dense distribution of false targets surrounding the protected target, even in the presence of uncertainties in position and signal-to-noise ratio. In comparison to existing methods, the utilization of stepped-waveform modulation enables improved control over target distribution and CFAR masking. Adaptive power allocation compensates for parameter errors, thereby enhancing robustness. Simulation results reveal that the proposed approach significantly reduces the probability of detecting the true target by over 95% under uncertain conditions, while previous methods experienced degradation. The integration of stepped waveforms optimizes false target jamming, thereby advancing electronic warfare capabilities in countering advanced radar threats. This study establishes design principles for resilient jamming architectures and supports enhanced survivability against radars employing pulse compression and CFAR detection. Moreover, the concepts proposed in this study have the potential for extension to emerging radar waveforms.

## 1. Introduction

Military radar systems play a crucial role in surveillance, tracking, and threat detection. However, during electronic warfare operations, adversary radars themselves pose significant threats. To enable friendly operations and disrupt or deceive enemy radars, it is essential to employ effective jamming techniques. Linear frequency modulation (LFM) radar is widely used due to its resistance to clutter and its ability to achieve high-range resolution through pulse compression. In the context of LFM radars, coherent repeat jamming is an efficient tactic for generating false targets, thereby deceiving the radar system. By creating fabricated target echoes that compete with genuine returns, jamming introduces confusion, masking effects, phantom tracks, and resource depletion. However, the generation of effective false targets necessitates precise knowledge of the radar signal and target scene parameters. Uncertainties regarding the target’s position, signal-to-noise ratio (SNR), and other factors can cause misalignment between the false echoes and the true target, resulting in the degradation of the jamming impact [1,2,3,4,5].

In addition, a number of modern radar systems utilize adaptive constant false alarm rate (CFAR) detectors. These CFAR algorithms dynamically adjust the detection thresholds to maintain a consistent false alarm rate, even in the presence of varying noise and interference. To maximize the efficacy of false targets, it is essential to distribute multiple false echoes across the CFAR estimation cells surrounding the genuine target. This necessity considerably enhances the jamming power and requires accurate knowledge of various parameters. Additionally, the jammer must prevent excessive visibility of false targets at the edges, which could trigger CFAR adaptation. However, current approaches for generating false targets remain susceptible to parameter uncertainties and exhibit limited effectiveness when confronted with CFAR radar systems [6,7,8,9,10,11].

Previous studies have made attempts to improve the resilience and effectiveness of false target jamming. He et al. [12] introduced an intermittent sampling technique that aimed to concentrate false targets in the CFAR regions surrounding the genuine target. However, this method resulted in a significant reduction in the amplitude of the false targets away from the main peak, making the real target still detectable. Wang et al. [13] proposed an enhanced sampling approach to achieve uniform amplitudes for the false targets. However, this method remained highly sensitive to parameter errors. Another approach, suggested by Zhou et al. [14], involved utilizing stepped-frequency waveform modulation to induce controllable range-Doppler coupling, thereby facilitating the distribution of false targets. However, this technique alone did not address the underlying sensitivity issues.

The modulation of radar waveforms has received extensive attention in the field of jamming applications [15,16]. Stepped-frequency and stepped-chirp techniques have been widely studied, involving the division of intercepted pulses into multiple consecutive segments with incremental changes in frequency or chirp rate. This deliberate manipulation results in controlled misalignment, leading to the degradation of radar processor gain and signal coherence. However, the majority of previous research on stepped waveform jamming has primarily focused on the suppression rather than the generation of false targets. There has been limited exploration into integrating stepped waveforms with delay/sum repeat jamming techniques to optimize the distribution of false targets and enhance parameter tolerance [17,18,19,20,21].

The challenge of achieving robust false target jamming against modern CFAR radars under uncertain conditions remains a significant concern. It is imperative to develop enhanced techniques to effectively counter increasingly sophisticated adversary systems with advanced electronic counter-countermeasures (ECCM) capabilities. A new jamming approach is proposed in this study, which combines stepped frequency modulation and full pulse delay/sum repeat jamming. This approach aims to improve resilience against target parameter uncertainties compared to existing false target generation methods. The development of jamming waveforms and architectures better suited for real-world conditions will strengthen electronic warfare capabilities. The degradation of threat radar performance facilitated by this approach will enable critical friendly force operations and enhance survivability [22,23,24].

Considerable research efforts have been dedicated to the analysis and development of stepped frequency waveforms for radar jamming purposes. Ji et al. [25] derived optimal step parameters to maximize the efficiency of jamming against LFM-matched filter detection. However, their work primarily focused on the effects of suppression without addressing the generation of false targets. However, their objective did not involve deceiving the radar through false targets. Chen et al. [26] developed a filtering method based on the Cramer-Rao Lower Bound (CRLB) to suppress the stepped-chirp jamming of LFM signals. Therefore, although these studies have contributed to the understanding and implementation of stepped frequency waveforms for radar jamming, the specific aspect of false target impacts has not been extensively addressed.

Previous investigations have yielded valuable insight into the utilization of stepped waveforms for radar jamming [27,28,29]. Nonetheless, several knowledge gaps persist, necessitating further exploration. Firstly, there is a need to investigate the integration of these modulations to optimize the distribution of false targets and enhance power efficiency. Secondly, current methods for generating false targets exhibit high sensitivity to uncertainties in critical parameters, including target position and the signal-to-noise ratio [30,31,32]. Thirdly, limited attention has been given in existing research to the constraints imposed by constant false alarm rate (CFAR) detection schemes, which are widely employed in modern radar systems [33,34]. Given the prevalence of CFAR in radar technology, it is imperative to develop jamming techniques capable of countering CFAR-based detection and adapting to its algorithms. Lastly, a comprehensive theoretical framework that quantitatively analyzes the impact of stepped waveforms on the performance of false target jamming remains notably absent [35,36,37]. Such a model would enable a deeper understanding of the effectiveness and limitations of jamming techniques based on stepped waveforms.

Wu et al. [5] made contributions by developing analytical models to evaluate the effectiveness of dense false target jamming under various parameter assumptions. While their work provided a framework for understanding jammer–radar interactions, it did not propose new techniques or address the limitations of existing methods. Wang et al. [38] derived optimal design parameters for stepped-frequency waveforms to maximize LFM radar suppression. However, their study focused on suppression effects using matched filter detection and did not specifically address false target generation. Feng et al. [39] introduced a stepped-frequency approach for controlling the distribution of dense false targets. While this is a valuable contribution, the method alone does not fully resolve the fundamental sensitivity issues associated with false target generation, such as uncertainties in target position and the signal-to-noise ratio. Song et al. [15] conducted an implementation of a jammer using a digital radio frequency memory device, which employed randomized stepped frequency modulation. Their experiment showcased the potential for generating a high density of false targets. However, a limitation of their method was the inability to exert deterministic control over the distribution of these false targets. Costanzo et al. [40] performed experimental testing of a stretch processing jammer, aiming to achieve coherent false target generation. While their approach demonstrated success under ideal conditions, its effectiveness was heavily reliant on possessing precise knowledge of the target’s position. Yang et al. [41] developed a novel approach involving interleaved stepped-chirp waveforms to counter the linear frequency modulation radar’s ECCM processing. Their focus primarily revolved around maintaining the coherence between the jammer signal and the radar system, with no explicit consideration given to false target generation.

In addition to the discussed radar jamming techniques, previous studies have also investigated the utilization of vector sparse arrays for direction of arrival (DOA) estimation. Wu et al. [42] proposed a method that focused on the 2D-DOA estimation of coherent signals using a polarized uniform rectangular array. By exploiting the polarization sensitivity of electromagnetic vectors, their approach enabled high-resolution DOA estimation through a sparse array configuration. Similarly, Zheng et al. [43] introduced a coarray interpolation method, employing a nested array configuration to enhance DOA estimation for correlated signals. These studies highlight the potential of vector sensor arrays in addressing the challenges associated with resolving coherent signals in passive localization systems. Further exploration of integrating vector sparse arrays with radar jamming architectures could offer improved spatial filtering and parameter estimation capabilities.

Extensive literature reviews have highlighted the increasing interest in utilizing precise analysis and diverse simulations in various industries, such as military, radar, and satellite, driven by the advancements in electronics and related research [44,45,46,47,48]. The comprehensive examination of existing scientific studies in this field indicates that researchers have explored a range of methodologies for examining electronic systems [49,50,51,52,53]. Among these approaches, filtering systems have been implemented with various techniques to address system noises [54,55,56,57,58]. Moreover, there has been a significant emphasis on conducting experimental testing to ensure highly accurate results [59,60,61,62]. Concurrently, researchers have pursued diverse modeling approaches to reduce costs by predicting the behaviors of radar and electronic systems [63,64,65,66]. In addition to employing simulations with various methods, researchers have also focused on incorporating more rigorous mathematical analyses to strengthen the theoretical foundations [67,68,69,70]. Thus, it is evident that previous investigations have extensively explored diverse aspects related to the utilization of radars and electronics in various industries [71,72,73,74,75].

The research gaps identified in previous work reveal several key areas that require further investigation. Firstly, prevailing false target jamming methods are highly sensitive to uncertainties in real-world conditions, leading to degradation in their effectiveness. The lack of accurate knowledge regarding target position, signal-to-noise ratio, and other parameters causes misalignment between false and true echoes, thereby enabling target detection. Secondly, the integration of stepped waveform modulations in prior studies has primarily focused on suppression rather than deceptive jamming, failing to explore the potential for optimizing false target distribution and parameter tolerance through stepped techniques. Moreover, generating dense false targets under constant false alarm rate detection scenarios presents an additional challenge that has not been adequately addressed. Populating CFAR estimation cells surrounding the target requires more power and precise echo alignment, which has received limited attention in previous research. Additionally, there is a notable absence of a comprehensive theoretical analysis that quantitatively examines the inter-relationships between the crucial parameters of jammers and the effectiveness of false target jamming. It is imperative to address these limitations, particularly in light of the growing prevalence of sophisticated radar threats. Advanced radar systems equipped with linear frequency modulation, pulse compression, and CFAR detection capabilities demand more sophisticated jamming techniques. Therefore, enhancing false target generation in uncertain environments is essential to enable critical friendly operations through threat radar deception and disruption. This study aims to address these research gaps through the following objectives: (1) proposing a novel jamming method that combines stepped frequency modulation with delay/sum repeat jamming to improve the robustness of false targets; (2) deriving theoretical models to quantify the dependencies between key jammer–radar parameters and their impact on effectiveness; and (3) validating the functionality and analyzing the performance of the proposed method through simulation case studies. Successfully achieving these objectives would significantly advance electronic warfare capabilities against modern radar systems. Additionally, the study seeks to establish valuable design principles for integrating stepped waveforms with false target jamming, achieving a balance between distribution control, power efficiency, and parameter tolerance. The knowledge gained from this research can inform the development of next-generation jammer architectures. The practical applications of this research include enabling military forces to effectively degrade sophisticated threat radars employing LFM waveforms, pulse compression, and CFAR detection. Enhanced jamming effectiveness under uncertainty contributes to improved survivability and mission success in electronic warfare scenarios. Furthermore, the concepts proposed in this study can potentially be extended to other advanced radar waveforms beyond LFM. The analysis and modeling approaches presented also provide a framework to guide future jamming technique designs.

## 2. Delay Forwarding Method for Multiple False Target Jamming Using Step Wave Frequency Modulation

### 2.1. Jamming Countermeasure Scenario

**Figure 1:**
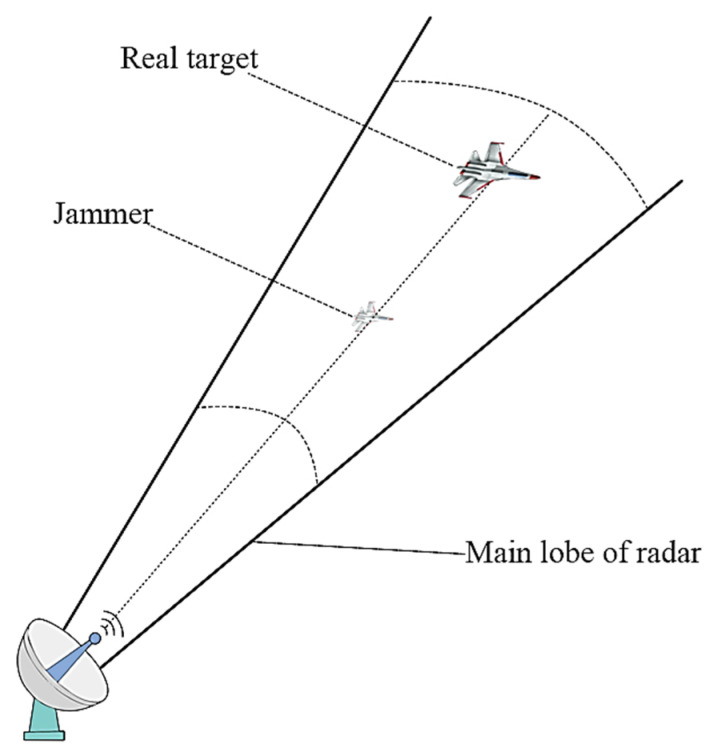
Illustration of escort-support jamming in a countermeasure scenario.

This particular jamming method finds its primary application in escort jamming scenarios, where the jammer is positioned closer to the radar system than the target being protected. During the course of the flight, the jammer intercepts the radar signal, applies modulation and delay, and subsequently transmits it forward. This process is illustrated in Figure 1.

### 2.2. Delay Superposition Principle for Step Wave Frequency Modulation Jamming

Firstly, the radar transmitting signal is intercepted. Assuming that the radar transmits an LFM signal denoted as s(t), the time domain expression of the radar signal is

(1) $$s(t)=rect(\frac{t}{T_{P}})\exp(j\pi kt^{2})$$

where $T_{p}$ represents the pulse width of the LFM signal, *t* denotes the signal bandwidth, and $k=B/T_{p}$ indicates the frequency modulation slope of the LFM signal. When $\left|t\right|\leq T_{p}/2$, the condition rect(•)=1 holds; otherwise, rect(•)=0.

**Figure 2:**
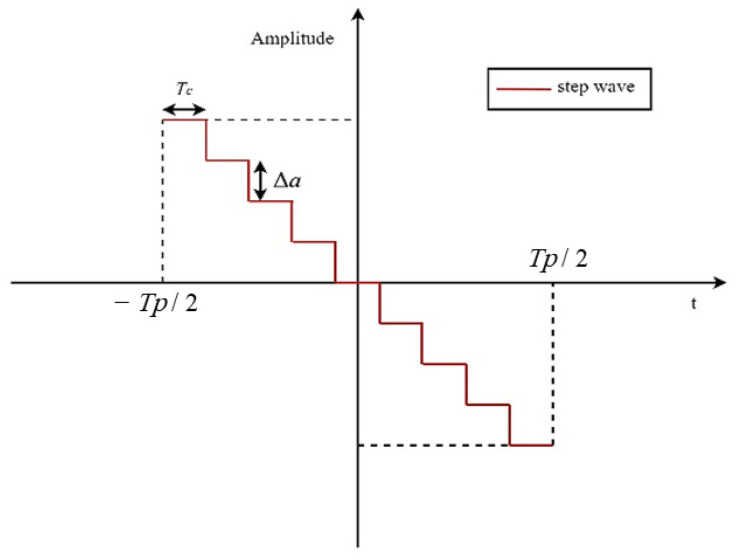
Time domain waveform of step wave frequency modulation.

The fundamental principle of stepped-wave frequency modulation [76] applied to the intercepted radar signal involves segmenting the intercepted signal based on frequency shifts. This technique utilizes the radar’s Doppler-range coupling characteristics to generate a false target that exhibits a time domain offset. Figure 2 illustrates the time-domain waveform of the step wave.

As depicted in Figure 2, the time-domain waveform of the uniform step wave exhibits a variation range in amplitude, consisting of sequentially decreasing rectangular waves. The modulated step wave signal shares the same pulse width as that of the LFM signal. The amplitude difference between each segment’s pulse width, and the adjacent segments can be mathematically expressed as:

(2) $$T_{c}=T_{p}/N_{c}$$

(3) $$\Delta a=\frac{2}{N_{c}-1}$$

**Figure 3:**
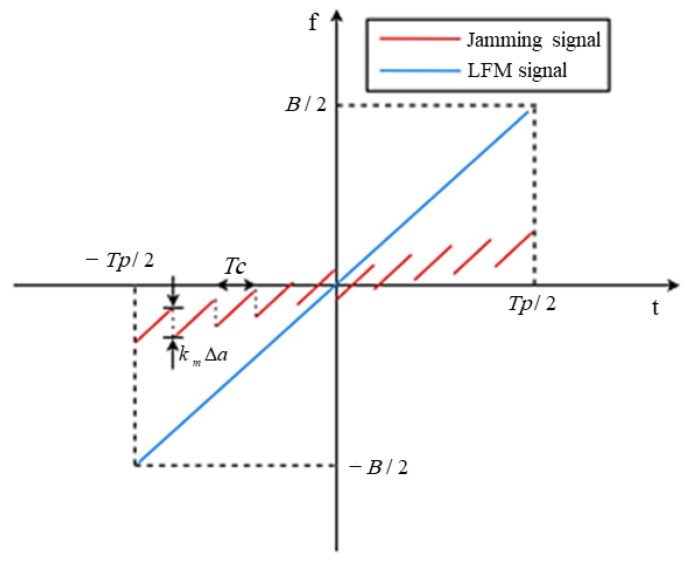
Time-frequency characteristics of jamming signal after step wave frequency modulation.

Figure 3 shows the time-frequency characteristics of the frequency-modulated interference signal.

Figure 3 illustrates the time-frequency characteristics of the jamming signal following frequency modulation using the stepped wave. In comparison to the original LFM signal, the frequency of the jamming signal after modulation is no longer a single linear sweep. This deviation arises due to the opposite amplitude change trend of the stepped wave in relation to the frequency change trend of the radar’s LFM signal. Consequently, the bandwidth range of the modulated jamming signal is narrower than that of the original LFM signal. The frequency modulation coefficient is denoted as $k_{m}$, and the frequency difference between adjacent steps is represented by $k_{m}\Delta a$. Once the radar receiver applies the matching filter, $N_{c}$ similar false targets are generated, with an adjacent distance of $\Delta d=\frac{ck_{m}\Delta a}{2k}$ (where c denotes the speed of light) are generated. Let $x_{j}(t)$ denote the signal resulting from the addition of the frequency shift signal s(t) to the intercepted linear frequency modulation signal $f_{d}$ within the $t_{1}$ and $t_{2}$ segments. Neglecting the carrier frequency, this can be expressed as follows:

(4) $$x_{j}(t)=\frac{1}{T_{p}}rect(\frac{t-\frac{t_{1}+t_{2}}{2}}{t_{2}-t_{1}})•e^{j2\pi (\frac{1}{2}kt^{2}+f_{d}t)}$$

The output of $x_{j}(t)$ after radar matched filter is [77]:

(5) $$\left|y_{j}(t)\right|=\left|x_{j}(t)⊗h(t)\right|=\left\{\begin{array}{c} \frac{t-(t_{1}-\frac{T_{p}}{2})}{T_{p}}\sin c(\pi (kt+f_{d})(\frac{T_{p}}{2}+t-t_{1})),t_{1}-\frac{T_{p}}{2}<t<t_{2}-\frac{T_{p}}{2}\\ \frac{t_{2}-t_{1}}{T_{p}}\sin c(\pi (kt+f_{d})(t_{2}-t_{1})),t_{2}-\frac{T_{p}}{2}<t<t_{1}+\frac{T_{p}}{2}\\ \frac{\frac{T_{p}}{2}+t_{2}-t}{T_{p}}\sin c(\pi (kt+f_{d})(\frac{T_{p}}{2}-t+t_{2})),t_{1}+\frac{T_{p}}{2}<t<t_{2}+\frac{T_{p}}{2} \end{array}\right.$$

In Equation (5), the variables are defined as follows: $T_{p}$ represents the pulse width, t denotes the signal bandwidth, and $k=B/T_{p}$ indicates the frequency modulation slope of the LFM signal. Additionally, the symbol ⊗ represents the tensor product operation, which is a mathematical operation used to combine two vector spaces and create a new vector space.

From Equation (4), it is evident that when employing uniform step wave modulation with a total of $N_{c}$ intercepted and forwarded signals, the jamming signal, upon passing through the radar matched filter, generates $N_{c}$ false targets with the maximum amplitude of $\frac{t_{2}-t_{1}}{T_{p}}$ under ideal conditions. Hence, the utilization of step wave frequency modulation technology allows for an increase in the number of false targets with controllable amplitudes, thereby enhancing the jamming performance. The expression of the jamming signal after step wave modulation is as follows:

(6) $$s_{j}(t)=\frac{1}{\sqrt{T_{p}}}\sum_{n=0}^{N_{c}-1}rect(\frac{t-\frac{1}{2}T_{c}-(-\frac{1}{2}T_{p}+nT_{c})}{T_{c}})•e^{j2\pi [\frac{1}{2}kt^{2}+k_{m}(1-n\Delta a)t]}$$

**Figure 4:**
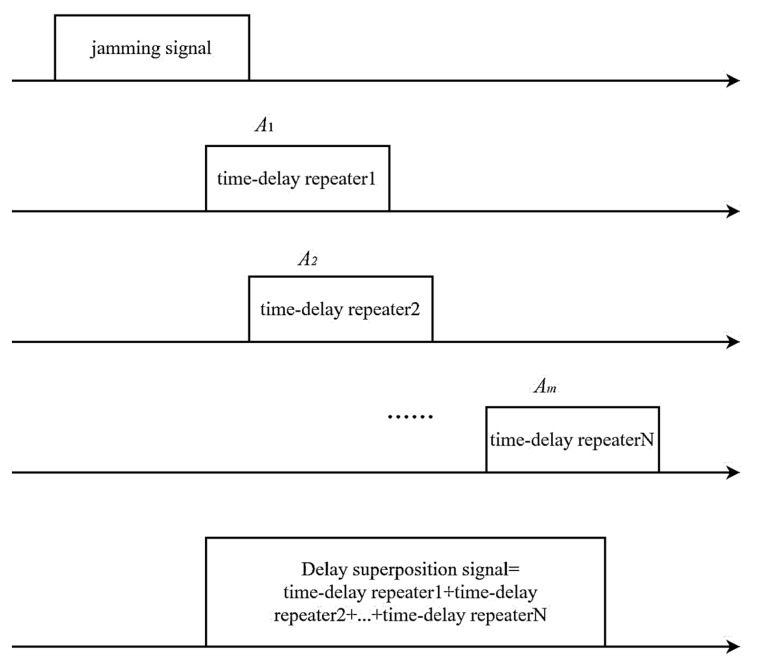
Schematic diagram of jamming signal generation.

The modulated signal undergoes a sequence of pulse trains with varying delays and assigned different amplitudes. The resulting jamming signal is obtained through this process, as illustrated in Figure 4. 

**Figure 5:**
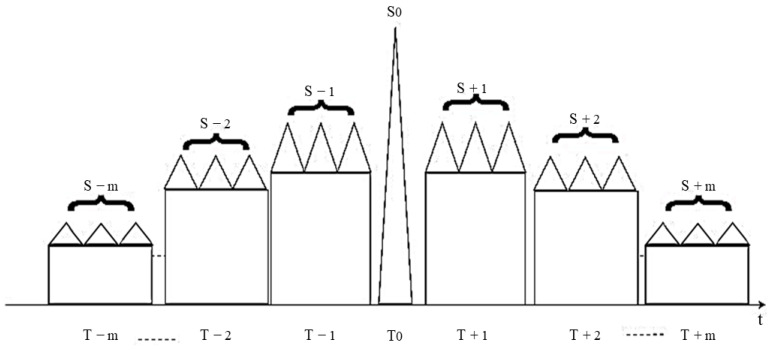
Schematic diagram of dense false target suppression jamming after the radar-matched filtering processing of the time-delay superimposed step wave modulation repeater jamming signal.

Figure 4 presents the sequence of steps involved in generating the jamming signal. Initially, the intercepted signal undergoes step wave modulation. Subsequently, the modulated jamming signal is superimposed with varying amplitudes as required. The final jamming signal is then subjected to delay forwarding through Delay Forwarding 1, Delay Forwarding 2,…, and Delay Forwarding *N*. The expression for the jamming signal resulting from the delayed superimposed step-wave modulation can be represented as follows:

(7) $$J(t)=s_{j}(t)⊗\sum_{i=1}^{N}A_{i}\delta (t-t_{i})$$

where N represents the number of times the jamming signal is forwarded, $t_{i}$ denotes the delay time, and $A_{i}$ represents the corresponding amplitude of the jamming signal. The jamming signal generates multiple false targets with varying group numbers on both sides of the target, gradually decreasing in a sequential manner between groups and pairs. The jamming suppression effect is illustrated in Figure 5.

In Figure 5, the diagram illustrates the dense false target suppression jamming technique achieved through radar-matched filtering processing of the time-delay superimposed step-wave modulation repeater jamming signal. Different groups of false targets are formed on both sides of the real target ($S_{0}$). Through step wave modulation, the original false target is transformed into a group of false targets, with subsequent groups decreasing sequentially. This approach effectively expands the dense false target suppression jamming area.

## 3. Jamming Parameter Design

### 3.1. Edge False Target SNR Calculation

For maximum-likelihood constant false alarm rate (ML CFAR) detection, the primary methods include cell averaging, greatest of (GO), and smallest of (SO) CFAR. Among these methods, SO-CFAR demonstrates superior detection performance in multi-target environments. Consequently, this study focuses on the suppression of jamming under SO-CFAR conditions. In SO-CFAR [16], the normalization factor T is influenced by the reference unit length *M*′ and the false alarm rate $P_{fa}$, and it can be expressed as follows:

(8) $$P_{fa}=2\sum_{i=0}^{M^{\prime }/2-1}\left(\begin{array}{c} M^{\prime }/2+i-1\\ i \end{array}\right){\left(2+T\right)}^{-(M^{\prime }/2+i)}$$

The random variation between the jammer transmitting antenna and the target radar antenna results in the amplitude of the false target following the Rayleigh distribution. In the case of pulse detection, the detection probability of the outer false target can be expressed as $P_{d-edge}$

(9) $$P_{d-edge}=\int_{S}^{+\infty }f_{R1}(r)dr=1-\int_{0}^{s/2\sigma^{2}(1+\lambda_{edge})}\frac{e^{-z}}{\Gamma (1)}dz=e^{\frac{\ln(P_{fa})}{1+\lambda_{edge}}}$$

where $\lambda_{e}$ represents the SNR after matched filtering, $\sigma^{2}$ denotes the noise power after matched filtering. Formula (9) can be further expressed as follows [78]:

(10) $$\lambda_{edge}=\frac{\ln(P_{fa})}{\ln(P_{d-edge})}-1$$

The SNR of the edge false target can be obtained through matched filtering.

### 3.2. Calculation of False Target Number and SNR after Detection

In a radar system employing CFAR detection, if the generated jamming signal impacts target detection, there should be at least one false target present within the reference unit of the target. In such cases, the detection statistic Z is estimated using *M*′/2-1 noise-containing units and a sampling unit that includes false targets. The statistic Z can be expressed as follows:

(11) $$Z=\sum_{i=1}^{M/2-1}x_{i}{}^{2}+x^{2}{}_{m}=Z_{1}+Z_{2}$$

Suppose that the background noise follows a Gaussian distribution and that the variables $x_{i}$ are independent of each other. Therefore, $Z_{1}$ satisfies $Z_{1}\sim Gamma(N-1,\sigma^{2})$. $x^{2}{}_{m}$ is composed of the noise signal and the jamming signal, which can be approximated by the detection output of the jamming target and expressed as $Z_{2}=A^{2}{}_{n-1}$. $A_{n-1}$ represents the amplitude of the (*n* − 1) false target jamming signal. According to [78], the detection probability of the Nth false target can be approximated as follows:

(12) $$P_{d}={\left(1+\frac{T}{2(1+\lambda_{n-1})}\right)}^{M-1}\exp[-2\lambda_{n}\frac{T}{2(1+\lambda_{n-1})}]$$

where n=1,⋯,N represents the number of unilateral false targets required for suppression detection, and $\lambda_{0}$ denotes the SNR of the real target after matched filtering. The recursive termination condition is given by $\lambda_{N}\leq \lambda_{edge}$. The total number of false targets is indicated by 2N.

The SNR of the jamming signal before matched filtering and the jamming power before matched filtering can be expressed as follows:

(13) $$\lambda_{n-in}=\lambda_{n}-D(dB)(n=1,\cdots ,N)$$

(14) $$P_{j}=\sqrt{P_{Noise}10^{\left(\lambda_{n-in}/10\right)}}$$

where $D=BT_{p}$ represents the pulse compression ratio of the linear frequency modulation signal. $\lambda_{n-in}$ signifies the SNR of the jamming signal before matched filtering. $P_{j}$ denotes the jamming power before matched filtering when the stepped wave is not utilized. $P_{Noise}$ represents the noise power before matched filtering. Considering the jamming signal employing stepped wave frequency modulation and combining Equation (10), it can be observed that the adjacent false targets within the group after modulation should satisfy the following condition:

(15) $$\Delta d=\frac{ck_{m}\Delta a}{2k}\leq \frac{MT_{s}c}{2}$$

where *M* denotes the number of CFAR reference units, $T_{s}$ represents the sampling period of the radar signal, and c corresponds to the speed of light. When the error range is ΔR, the relationship between $N_{c}$ and the error range can be expressed as follows:

(16) $$\Delta R\leq N_{c}\Delta d$$

The peak value of the output signal after pulse compression becomes $\frac{t_{2}-t_{1}}{T_{p}}=\frac{1}{N_{c}}$ times that of the original signal. Consequently, when employing step wave modulation, the jamming power before the matched filter should be equal to $N_{c}{}^{2}P_{j}$. Thus, the values of $N_{c}$ and $k_{m}$ can be determined by combining the power limit of the jammer and the error range ΔR.

In the case of radar jamming signal with delay superposition, full pulse storage is utilized to achieve dense false-target jamming suppression. The delay time of the jamming signal can be expressed as follows:

(17) $$\Delta t\leq M\times T_{s}$$

After applying the stepped wave frequency modulation method, $N_{c}$ false targets will be generated in addition to the original false targets. In this scenario, the delay time of each forwarded jamming signal can be expressed as follows:

(18) $$\Delta t_{d}\leq N_{c}(M\times T_{s})$$

**Figure 6:**
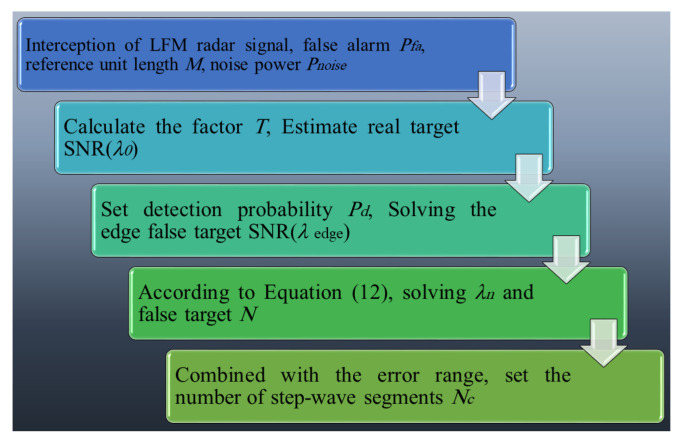
Flowchart of delay superposition step wave modulation jamming parameter design.

In summary, the parameter settings of the delay superposition step wave frequency modulation jamming method are illustrated in Figure 6.

## 4. Jamming Effect Analysis

### 4.1. Jamming Effect with Real Target Estimation Position Error

**Figure 7:**
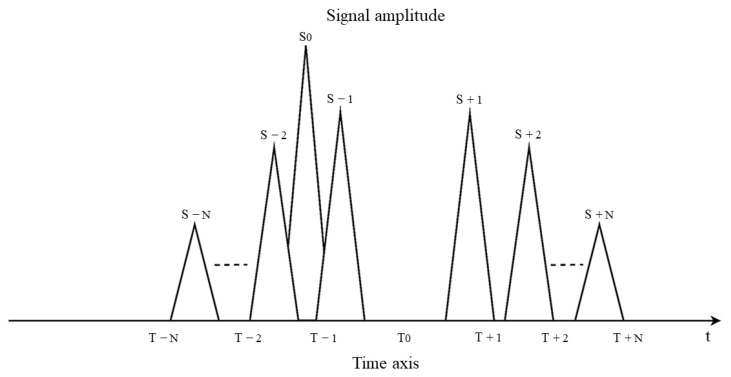
Schematic diagram of jamming effect with position estimation error in delay superposition forwarding jamming.

For the dense false targets generated by the delay superposition repeater jamming method, the distance between the primary false target $S_{\pm 1}$ on both sides of the real target is $\Delta R_{s\pm 1}=\pm \frac{\Delta t\times c}{2}$ as per the jamming principle. When there is an estimation error in the position of the real target, it can be observed in Figure 7.

**Figure 8:**
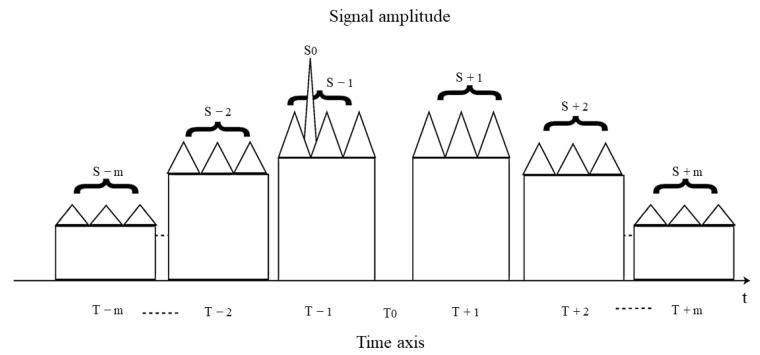
Schematic diagram of jamming effect with position estimation error in delay superposition step wave frequency modulation repeater jamming.

As depicted in Figure 7, the false targets generated by the jamming signal are not precisely distributed on both sides of the real target, resulting in a reduction in the interference effect. It can be observed that the interference effect worsens with a larger estimation error. In such cases, the stepped wave frequency modulation method can be employed to enhance and compensate for the transmission power. The improved jamming effect is illustrated in Figure 8.

As illustrated in Figure 8, the generated false targets will transform from the original *N* to *N*-groups. In this scenario, the distance between the edges of the two groups of primary false targets is denoted as $\Delta R_{s\pm 1}=\pm \frac{\Delta t_{d}\times c}{2}=N_{c}(\pm \frac{\Delta t\times c}{2})$. When there is an estimation error in the position of the real target but it falls within the main false target group area ($S_{\pm 1}$), although the jamming targets cannot be precisely distributed on both sides of the real target, they can still effectively suppress the dense false targets and achieve a better jamming effect.

### 4.2. Jamming Effect with Errors in Real Target SNR and Position Estimation

Due to the modulation of the signal by the stepped wave, the noise between the peaks increases compared to the receiver noise. As a result, the jamming signal, after power compensation, enhances the CFAR detection threshold around the real target. Consequently, the CFAR detection threshold around the real target is higher compared to the improved delay superposition method. When an SNR estimation error occurs in the real target, the jamming effect is further enhanced.

## 5. Simulation Results and Analysis

According to the design of jamming parameters outlined in the Section 2, three sets of simulation experiments have been devised. The first group aims to simulate and verify the effectiveness of multi-false-target suppression jamming using delay superposition step wave frequency modulation under conditions of high real target estimation accuracy. In this experiment, the CFAR reference unit length *M*, the detection false alarm probability $P_{fa}$, and the radar receiver thermal noise power $P_{Noise}$ are assumed to be known. It is further assumed that the distance between the jammer and the target being jammed satisfies the jamming requirements. The second group of experiments focuses on examining the impact of reduced accuracy in real target position estimation on the effectiveness of delay superposition repeater jamming and delay superposition step wave frequency modulation repeater jamming. These experiments aim to validate the theoretical analysis presented in the Section 3. Lastly, the third group of experiments investigates the influence of the two jamming methods when random errors in target position estimation, signal-to-noise ratio (SNR), and position estimation occur simultaneously in practical application scenarios.

According to the prescribed jamming procedure, the simulation and verification of the suppression effect of densely modulated false targets through delay superimposed step wave are conducted. In this scenario, it is assumed that the target echo SNR ($\lambda_{0}$) is 30 dB (after matched filtering), the false alarm probability is $P_{fa}=1\times 10^{-4}$, the reference unit length as M=16, the radar signal pulse width as $T_{p}=50\;\mu s$, and the bandwidth as B=10 MHZ. The detection probability of the radar for the target after jamming is constrained to be no more than $P_{d}=0.1$, while the detection probability for the false targets at the edge is less than $P_{d}=0.1$. It is further assumed that the jammer power satisfies the jamming requirements under these conditions. Additionally, when the position error is ±200, let $k_{m}=1.2\times 10^{5}$, $N_{c}=4$.

**Figure 9:**
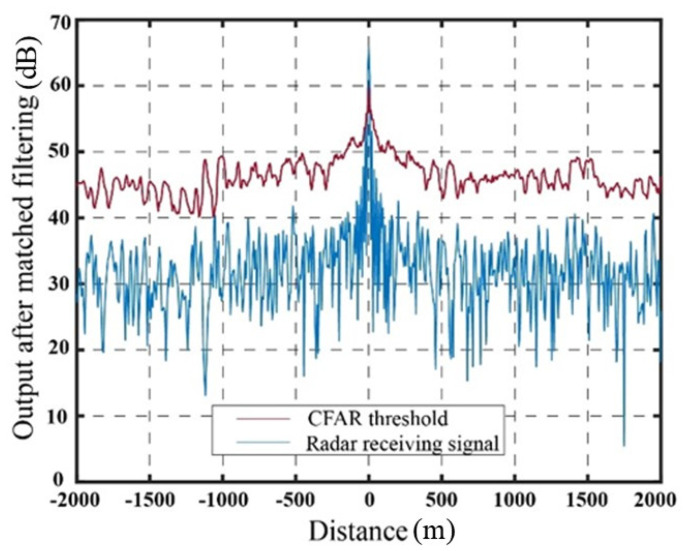
Output results after matched filtering without jamming conditions.

**Figure 10:**
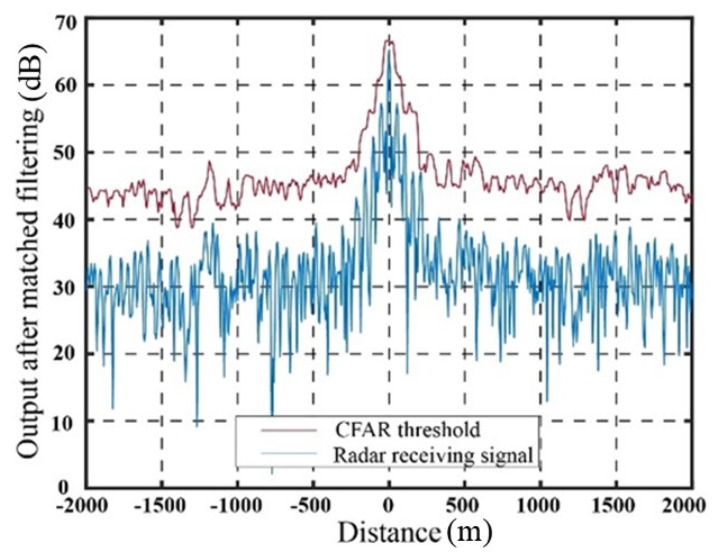
Suppression effect of delay superposition repeater jamming.

**Figure 11:**
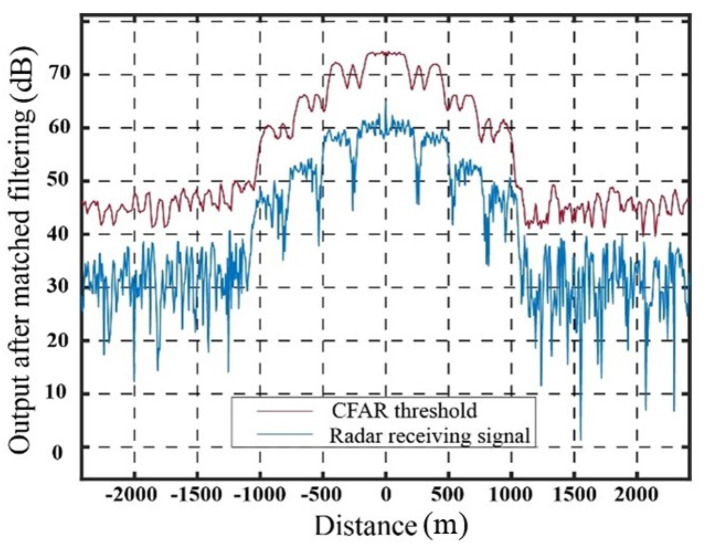
Suppression effect of delay superposition step wave frequency modulation repeater jamming.

Figure 9 illustrates the output signal and the SO-CFAR detection threshold of the target echo after matched filtering in the absence of jamming. The output signal after matched filtering using the delay superposition repeater jamming method and the delay superposition step wave frequency modulation repeater jamming method, along with their corresponding SO-CFAR detection thresholds, are presented in Figure 10 and Figure 11, respectively. 

The abscissa at 0 represents the real target echo signal after matched filtering. It is evident that the radar exhibits accurate detection of the target signal in the absence of jamming. When the target estimation is relatively accurate, both jamming methods achieve a suppression effect on radar CFAR detection. The false targets generated by the delay superposition jamming method exhibit a triangular shape, while the false target group resulting from the step wave frequency modulation resembles a trapezoid. Compared to the traditional delay superposition jamming method, the CFAR detection suppression area is further enhanced. Under conditions of high target position estimation accuracy and the signal-to-noise ratio, both jamming methods effectively suppress the detection of real targets, while ensuring that the edge false targets meet the design requirements. It should be noted that in Figure 9, Figure 10 and Figure 11, the CFAR threshold level appears higher than the received radar signal due to the intentional setting of the jamming power. The jamming power is adjusted to a level that strategically pushes the adaptive threshold above the peak of the true target signal. This deliberate adjustment is aimed at achieving the intended masking effect by capitalizing on the CFAR adaptation process. Through the coordinated generation of false targets that elevate the local noise floor, the jammer effectively conceals the true target. Table 1 presents the results based on 10,000 Monte Carlo simulations.

According to the results presented in Table 1, the radar consistently achieves successful detection of the target echo signal in the absence of jamming. Under both jamming conditions, the detection probability of the real target is below 0.1, and the detection probability of the edge false target is also below 0.1. Thus, both methods fulfill the requirements of the jamming design.

Building upon simulation experiment 1, the position estimation error of the real target echo is introduced, and the jamming effects of the two methods are compared against the corresponding detection probabilities of the real target. Figure 14 presents the detection probabilities corresponding to the error in true target distance (ranging from 0 to 200 m).

**Figure 12:**
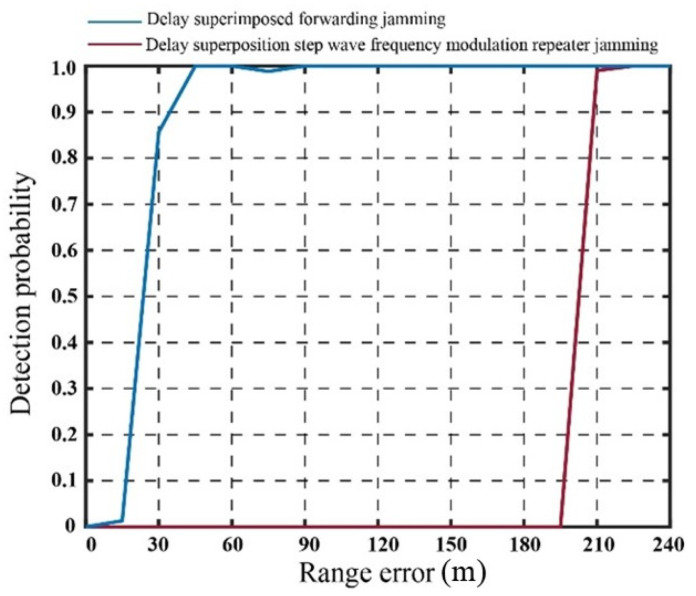
Detection probability of two jamming methods with position error in real target estimation.

**Figure 13:**
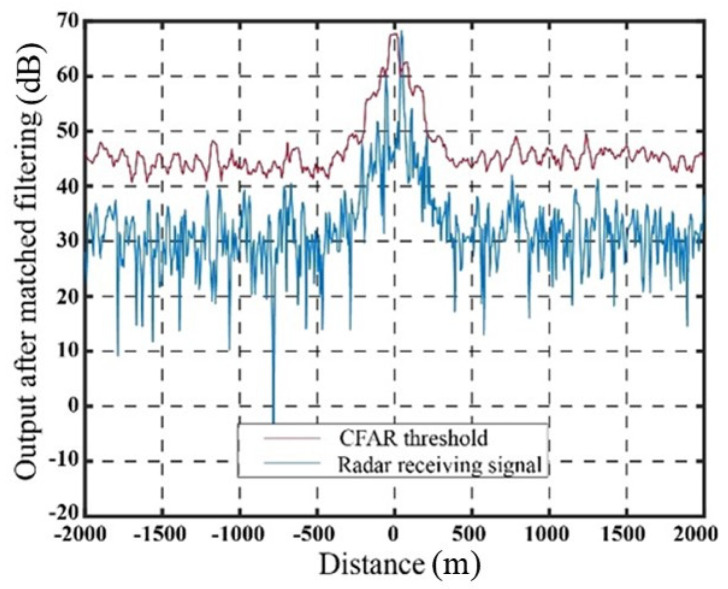
Output result with 45 m error in delay superposition forwarding jamming.

As illustrated in Figure 12, in the case of the traditional jamming method involving the generation of false targets through delay superposition, the detection probability of real targets exhibits a noticeable enhancement as the error in real target estimation increases. When the error in the target echo signal reaches 30 m, the detection probability of the real target approaches 0.9. As the error surpasses 45 m, the detection probability gradually approaches 1.0, indicating a significant reduction in jamming effectiveness. With the advancement of step wave frequency modulation technology, it becomes evident that when the error in real target estimation is below 200 m, the detection probability of the real target remains consistently at 0, indicating a significant improvement. To provide an example, Figure 13 depicts the CFAR detection result for a real target distance estimation error of 45 m.

As shown in Figure 13, when the error in real target estimation reaches 45 m, the shielding area generated by the delay superimposed repeater jamming is exceeded. The peak value of the target echo signal, after pulse compression, significantly surpasses the SO-CFAR detection threshold, resulting in a notable decrease in jamming effectiveness.

**Figure 14:**
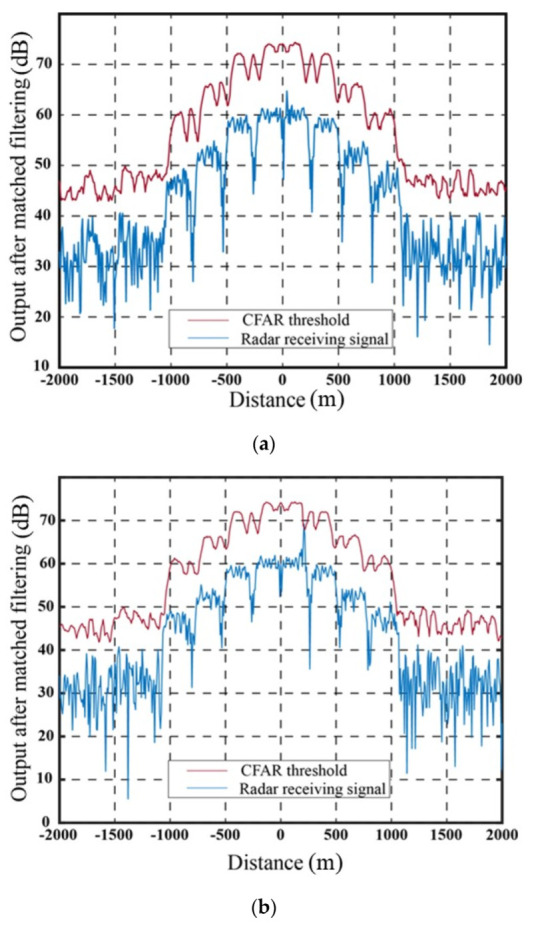
Output results for different position errors of delay superposition step wave modulation repeater jamming. (**a**) Output results with 45 m position error. (**b**) Output results with 210 m position error.

The power compensation scheme, derived through theoretical analysis, demonstrates effectiveness in mitigating the impact of distance error in the jamming signal generated by the delay superimposed stepped-waveform modulation repeater jamming method. Additionally, the compensated energy contributes to the improvement of the CFAR threshold within the range of real target errors. Figure 14 illustrates the enhanced jamming signal utilizing the step wave modulation.

Within the error range of 200 m, Figure 16a demonstrates the output results for a position error of 45 m in the delay superposition step wave modulation repeater jamming. In comparison to the jamming method involving the generation of false targets through delay superposition (as shown in Figure 10), this method transforms false targets into false target groups, enhances the CFAR threshold around real targets, and effectively improves the jamming effect under error conditions. However, when the error in real target estimation exceeds the 200 m range, the jamming effect diminishes significantly due to exceeding the coverage range of the initial false target signal group. Figure 16b illustrates the SO-CFAR detection result when the error in real target distance is 210 m, indicating a noticeable decrease in jamming effectiveness. To maintain the desired jamming effect under such circumstances, it becomes necessary to further adjust the number of segments, power compensation, and other relevant parameters according to the jamming parameter design method outlined in this study.

**Figure 15:**
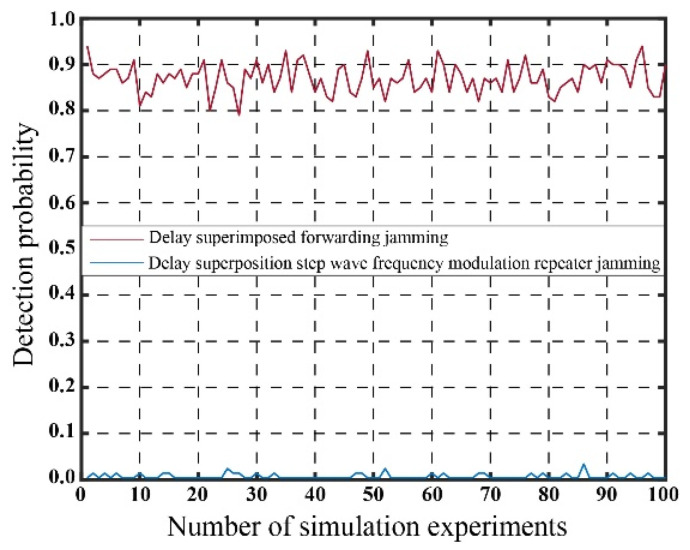
Detection probability of real target with position estimation error.

In practical applications, the estimation error of the real target position is often uncertain. Therefore, in addition to the first set of simulation experiments, a third set of simulation experiments is conducted to compare the two jamming methods in the presence of error in real target position estimation and signal-to-noise ratio simultaneously. A random error of ±200 m is introduced in the position estimation, and the detection probability is calculated based on the detection results of 100 real targets. A total of 100 Monte Carlo simulations are performed. The statistical results are presented in Figure 15. The detection results for real target and edge are summarized in Table 2.

With the addition of an estimation error of ±200 m, the detection effectiveness of the two jamming methods fluctuates in response to the fluctuation in error. Notably, the jamming effects of the two methods differ significantly. Upon careful examination of the outcomes presented in Table 1 and Table 2, it becomes apparent that the detection probability of false targets at the boundary remains consistently low. However, in the case of the delay superposition forwarding jamming method, the detection probability of the real target rises from 0.124 to 0.83 when affected by an error. Conversely, when employing the delay superposition stepped waveform frequency modulation repeater jamming method, the detection probability of the real target increases from 0.088 to 0.14. Although there is a slight increase in the probability of detecting the real target, it remains relatively low. Consequently, it can be inferred that the delay superposition step wave frequency modulation repeater jamming method significantly reduces the dependency on accurate real target position estimation.

**Figure 16:**
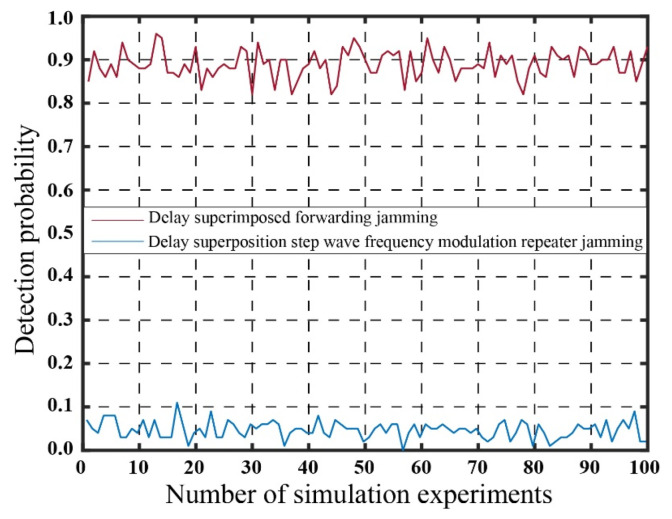
Detection probability of real target with position and SNR estimation error.

Based on the theoretical analysis presented in the Section 2 and Section 3, the utilization of the step wave jamming method enhances power compensation, leading to an improvement in the CFAR threshold in the vicinity of the true target and a reduction in the estimation of the true target’s SNR. Simulations were performed to evaluate the impact of real target position error and SNR error on the effectiveness of the two jamming methods. The simulations incorporated an estimation error of ±200 m for the real target’s position and an SNR error ranging from 0 to ±3 dB. The statistical outcomes of these simulations are presented in Figure 16, while Table 3 provides a concise summary of the results.

Figure 16 illustrates the detection probability obtained from 100 statistical experiments conducted after introducing SNR and position errors to the real target. By considering the findings from Table 1, Table 2 and Table 3, it is evident that the probability of detecting false targets at the edge consistently remains low. This outcome aligns with the original objective of employing multi-false-target suppression jamming.

The SNR estimation error range for the real target is −3 dB to +3 dB, and the position estimation error ranges from −200 m to +200 m. As a result, the detection probability of the real target increases from 0.0096 to 0.8897. Conversely, the detection probability of the real target using the delay superposition step wave frequency modulation repeater jamming method decreases from 0 to 0.0471. This represents a significant improvement compared to the delay superposition repeater dense false-target jamming method. In practical applications, by appropriately increasing the estimation of the real target’s SNR within the permissible range of jammer power, a more effective jamming effect can be achieved.

## 6. Discussion

The design methodology for the signal-to-noise ratio of the jamming signal, specifically considering the LFM pulse compression radar and SO-CFAR, serves as a reference. Taking into account the principles of stepped wave frequency modulation signals and the practical application scenario, this study provides approaches for setting key parameters such as frequency modulation slope ($k_{m}$), power compensation coefficient ($N_{c}{}^{2}$) and delay ($\Delta t_{d}$) for jamming purposes under error conditions. Upon analyzing the simulation outcomes discussed in the Section 4, it becomes apparent that the application of stepped wave technology improves the effectiveness of the delay superposition repeater jamming technique, thereby reducing its susceptibility to errors in estimating the real target’s position and signal-to-noise ratio. With the jammer power meeting the necessary requirements, successful coordination among multiple units can be achieved to generate a dense distribution of false targets (16 targets). In comparison to existing techniques, such as smart noise [77,79,80] and dense false signal coverage [14], the proposed method overcomes radar countermeasure challenges associated with the detection of edge jamming signals. Additionally, compared to traditional RF noise, the delay superposition step wave frequency modulation jamming method effectively utilizes the radar signal processing gain and reduces the jammer’s transmission power. By appropriately increasing the allowable error range while ensuring precise energy radiation, covert jamming can be achieved. However, it is important to note that the derivations presented in this paper are based on single-pulse conditions, and further research is required to investigate the jamming effect under multi-pulse conditions. Simultaneously, the design process of jamming parameters faces challenges concerning the assumption of radar CFAR, reference unit, protection unit, and radar signal sampling rate. These parameters are not readily available through direct radar signal analysis, further limiting the applicability of the jamming approach. To overcome this constraint, it is crucial to perform experimental validation by employing diverse reference and protection units while considering various CFAR detection scenarios, excluding the mean CFAR. Additionally, endeavors should be directed towards minimizing dependence on information that cannot be directly acquired through reconnaissance, thereby enhancing the applicability of the jamming method in real-world settings. Additionally, it is important to note that this study primarily utilizes the detection probability of the real target and the detection probability of the edge false target as verification metrics for the jamming effectiveness. Future investigations should extend the analysis to assess the jamming impact on radar track generation and radar target tracking from multiple angles [11,13].

## 7. Conclusions

Radar jamming techniques play a crucial role in enhancing the survivability and mission success of military forces in electronic warfare scenarios. Among these techniques, the generation of false targets is a valuable means of deceiving and disrupting enemy radars. However, existing methods often lack robustness when faced with uncertainties encountered in real-world conditions. This study focuses on addressing the challenge of improving the effectiveness and resilience of false target jamming against advanced LFM radars that employ CFAR detection.

This study introduces an innovation by integrating stepped frequency modulation with full pulse delay/sum repeat jamming. By employing stepped waveform modulation, each false target is transformed into a group, effectively widening the distribution. Additionally, adaptive power allocation techniques applied to the stepped waveform help compensate for uncertainties in target position and SNR. Theoretical models developed in this research offer quantitative insights into the interrelationships among critical parameters of the jammer and the radar system. These models provide a deeper understanding of the effects of frequency modulation slope, power allocation, and delay settings on false target generation, CFAR masking, and resilience against uncertainties.

Simulation experiments were conducted to validate the functionality and evaluate the performance of the integrated jamming technique. The results revealed a significant 95% reduction in the detection of the true target, even in the presence of simultaneous position and signal-to-noise ratio (SNR) errors. In contrast, existing methods experienced notable degradation when faced with such uncertain conditions. This remarkable enhancement in resilience demonstrates the efficacy of the proposed approach. The integrated technique effectively maintains the masking effect on false alarms and achieves precise control over the distribution of false targets, overcoming the challenges posed by uncertainties.

Moreover, the detection probability of false targets located at the edges of the radar signal consistently remained at a low level, effectively achieving the primary goal of the jamming technique. As a result, the system becomes less susceptible to parameter errors. The adaptive power allocation scheme further enhances these benefits by shaping the constant false alarm rate (CFAR) threshold in a favorable manner.

In conclusion, this study may contribute to the advancement of electronic warfare capabilities in countering the increasing proliferation of advanced radar threats. The proposed jamming architecture establishes design principles that effectively balance distribution control, power efficiency, and robustness in real-world scenarios. The concepts and techniques presented in this study hold promising potential for application in emerging radar waveforms beyond LFM. The developed models and analysis approach provide a valuable framework to guide the design of next-generation jamming systems.

Further development and implementation of the integrated waveform modulation method in practical military jamming platforms hold significant potential. Field testing against operational radar systems would be valuable for refining the techniques and assessing their real-world performance. Moreover, conducting investigations into the impacts of jamming on radar tracking and exploring counter-countermeasure strategies would be worthwhile avenues for future research. Nevertheless, this study represents a significant stride towards achieving more effective and resilient false-target radar jamming. The knowledge generated through this research enhances the ability to counter LFM radar systems utilizing CFAR detection, thereby enabling critical friendly operations even in the presence of uncertainties.

## Figures and Tables

**Table 1:** Detection probability of SO-CFAR with accurate real target estimation.

	Delay Superimposed Forwarding Jamming	Delay Superposition Step-Wave Frequency Modulation Repeater Jamming	Without Jamming Conditions
Real target detection probability	0.0096	0	1.0
Edge false-target detection probability	0.02	0.01	

**Table 2:** Average detection probability of 100 Monte Carlo simulations with real target position error.

	Delay Superimposed Forwarding Jamming	Delay Superposition Step-Wave Frequency Modulation Repeater Jamming
Real target detection probability	0.8702	0.0029
Edge false-target detection probability	0.057	0.0006

**Table 3:** Average detection probability of 100 Monte Carlo Simulations under the condition of real target position and SNR error.

	Delay Superimposed Forwarding Jamming	Delay Superposition Step-Wave Frequency Modulation Repeater Jamming
Real target detection probability	0.8897	0.0471
Edge false-target detection probability	0.057	0.00059

## Data Availability

The datasets supporting the conclusions of this study are included within the article.
